# Prognostic Value of the Advanced Lung Cancer Inflammation Index Ratio in Patients with Acute Myocardial Infarction Complicated by Cardiogenic Shock: A Cohort Study

**DOI:** 10.31083/j.rcm2507267

**Published:** 2024-07-18

**Authors:** Ming Gong, Bryan Richard Sasmita, Yuansong Zhu, Siyu Chen, Yaxin Wang, Zhenxian Xiang, Yi Jiang, Suxin Luo, Bi Huang

**Affiliations:** ^1^Department of Cardiology, The First Affiliated Hospital of Chongqing Medical University, 400016 Chongqing, China

**Keywords:** acute myocardial infarction, cardiogenic shock, advanced lung cancer inflammation index, short-term outcome

## Abstract

**Background:**

Acute myocardial infarction (AMI) complicated by cardiogenic shock (CS) carries a high mortality risk. Inflammation and nutrition are involved in the pathogenesis and prognosis of both AMI and CS. The advanced lung cancer inflammation index ratio (ALI) combines the inflammatory and nutritional status. Our present study aimed to explore the prognostic value of ALI in patients with CS following AMI.

**Methods:**

In total, 217 consecutive patients with AMI complicated by CS were divided into two groups based on the ALI admissions cut-off: ≤12.69 and >12.69. The primary endpoint of this study was 30-day all-cause mortality. The secondary endpoints were gastrointestinal hemorrhage and major adverse cardiovascular events (MACEs), including 30-day all-cause mortality, atrioventricular block, ventricular tachycardia/ventricular fibrillation, and nonfatal stroke. The association of ALI with the study endpoints was analyzed by Cox regression analysis.

**Results:**

During the 30-day follow-up period after admission, 104 (47.9%) patients died and 150 (69.1%) suffered MACEs. The Kaplan–Meier analysis revealed significantly higher cumulative mortality and lower MACE rates in the low-ALI group compared to the high-ALI group (both log-rank *p* < 0.001). The 30-day mortality rate was significantly higher in patients with ALI ≤12.69 compared to ALI >12.69 (72.1% vs. 22.6%; *p* < 0.001). Furthermore, the incidence of MACEs was higher in patients with ALI ≤12.69 (85.6% vs. 51.9%; *p* < 0.001). The receiver operating curve showed that ALI had a modest predictive value (area under the curve [AUC]: 0.789, 95% confidence interval [CI]: 0.729, 0.850). After multivariable adjustment, ALI ≤12.69 was an independent predictor for both 30-day all-cause mortality (hazard ratio [HR]: 3.327; 95% CI: 2.053, 5.389; *p* < 0.001) and 30-day MACEs (HR: 2.250; 95% CI 1.553, 3.260; *p* < 0.001). Furthermore, the addition of ALI to a base model containing clinical and laboratory data statistically improved the predictive value.

**Conclusions:**

Assessing ALI levels upon admission can provide important information for the short-term prognostic assessment of patients with AMI complicated by CS. A lower ALI may serve as an independent predictor of increased 30-day all-cause mortality and MACEs.

## 1. Introduction

Acute myocardial infarction (AMI) is one of the main causes of cardiogenic shock (CS) [1]. Furthermore, ongoing myocardial ischemia or infarction can result in left-ventricular dysfunction, reduced cardiac output, end-organ hypoperfusion, and hypoxia [1]. Despite early revascularization and aggressive treatment, the mortality rate of AMI complicated by CS remains high [1]. Atherosclerosis, often present in AMI, is characterized by a chronic, low-grade inflammatory response which recruits both innate and adaptive immune cells into the atherosclerotic plaque [2]. There is growing evidence suggesting that inflammatory reactions play a significant role in the development of atherosclerosis and adverse cardiac remodeling. Several inflammatory markers, such as C-reactive protein (CRP), neutrophil–lymphocyte ratio (NLR), leukocytes, and neutrophils, have been identified as important prognostic indicators in patients with AMI complicated by CS [3, 4, 5].

The advanced lung cancer inflammation index (ALI), initially developed to gauge inflammation in non-small cell lung cancer (NSCLC) integrates information about the inflammatory state and nutritional status [6]. However, subsequent studies have indicated that ALI could also be a prognostic indicator for other diseases, including multiple myeloma, heart failure (HF), and Crohn’s disease [7, 8, 9]. Despite its expanding application, there is a lack of data evaluating ALI as an inflammatory indicator or biomarker in patients with CS following AMI. Therefore, in the present study, we sought to investigate the impact of ALI on short-term outcomes in patients with CS following AMI.

## 2. Methods

### 2.1 Study Design and Participants

**Fig. 1.**
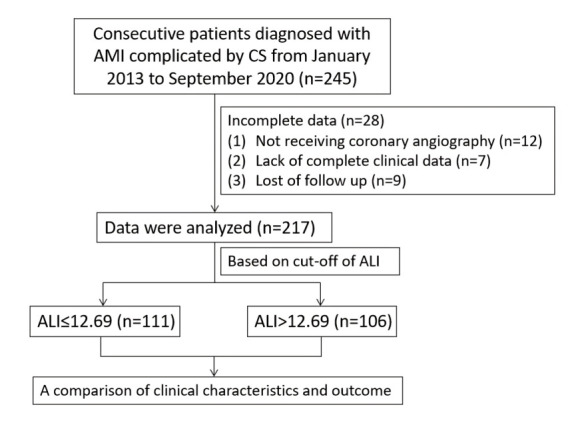
**Study flow chart. **AMI, acute myocardial infarction; CS, cardiogenic shock; ALI, advanced lung cancer inflammation index.

This is a retrospective analysis of 245 consecutive patients diagnosed with CS complicating AMI in our center from January 2013 to September 2020. Among the 245 patients, 28 patients had incomplete data or loss of follow-up, and the remaining 217 patients were included in this study (Fig. 1). This study was performed based on the principle of the Declaration of Helsinki and the study protocol was approved by the Institutional Ethical Review Board of The First Affiliated Hospital of Chongqing Medical University (No. 2020-233).

### 2.2 Definitions

AMI was diagnosed if there was a rise and/or fall of cardiac marker, troponin (cTn) values with at least one value above the 99th percentile upper reference limit with one of the following features: symptoms of ischemic chest pain, typical electrocardiography (ECG) presentations, evolution of pathological Q waves, and imaging evidence of new losing of viable myocardium or new ventricular regional wall motion abnormality consistent with the ischemic etiology [10]. If a patient experienced typical ischemic chest pain symptoms but had an atypical ECG presentation on ECG, especially if the time from symptom onset to admission was short and the cTn testing was negative, cTn testing was repeated within 1–3 hours of admission.

The definition of CS was a pathophysiological state in which cardiac output is reduced, leading to inadequate perfusion of the tissue [1]. The main diagnostic criteria of CS include (1) hypotension, (2) systolic blood pressure (SBP) <90 mmHg or the mean arterial pressure reduces more than 30 mmHg from baseline, (3) reduced cardiac index <2.2 [L/min]/$m^{2}$ body surface in patients who received vasoactive or mechanical support therapy, or <1.8 [L/min]/$m^{2}$ body surface in those who did not receive vasoactive or mechanical support therapy, and (4) the filling pressure was within normal range (pulmonary artery wedge pressure >15 mmHg) [11].

### 2.3 Managements

A comprehensive evaluation was provided for patients upon admission. Emergent coronary angiography was recommended to figure out the culprit artery, and if possible, percutaneous coronary intervention (PCI) was performed. Patients who had complex and severe lesions after coronary angiography with hypotension, an intra-aortic balloon pump was implanted. After the procedure, patients were sent to the intensive care unit for further monitoring, treatment, and management. Standard therapy for AMI such as antiplatelet drugs, β-blockers, angiotensin-converting enzyme inhibitors (ACEI)/angiotensin-converting receptor blockers (ARB), and other routine comprehensive treatments were administered according to the guidelines of the management of CS complicating AMI [11]. Meanwhile, the differential diagnosis of chest pain and CS such as aortic dissection, pulmonary embolism, and an acute decompensated chronic heart failure was performed based on the clinical symptoms, previous medical histories, physical examination, presentations on ECG, and myocardial biomarkers testing.

### 2.4 Data Collection

Baseline characteristics, including age, sex, clinical presentation, comorbidities, and medical history were extracted from the electronic medical system by experienced physicians and nurses. Echocardiography was examined within 24 h after admission. Blood samples were collected immediately upon admission and were tested in the central laboratory. Blood tests were performed by the Shanghai Sysmex XN-1000 automatic blood cell analyzer (Sysmex Corporation, Shanghai, China) and serum albumin was assessed using the liver function test. The formula for deriving the inflammatory index in advanced lung cancer is as follows: ALI = body mass index (BMI) × Alb/NLR, where BMI is the body mass index (${\mathrm{kg/m}}^{2}$), Alb is the serum albumin (g/dL), and NLR is the absolute neutrophil count/absolute lymphocyte count.

### 2.5 Endpoints

The primary endpoint of this study was 30-day all-cause mortality. The secondary endpoints were gastrointestinal hemorrhage and major adverse cardiovascular events (MACEs), including 30-day all-cause mortality, atrioventricular block, ventricular tachycardia (VT)/ventricular fibrillation (VF), and nonfatal stroke.

### 2.6 Statistical Analysis

Continuous data were expressed as means ± standard deviation (SD) or median and interquartile values based on the distribution and variance. Comparisons of baseline characteristics were performed with the Pearson χ^2^ test, Fisher exact test, or Mann–Whitney *U* test, as appropriate. The area under the curve (AUC) for the 30-day mortality and 30-day MACEs was calculated by the receiver operating characteristic (ROC) curve analysis to determine the predictive value of the ALI. Patients were then divided into two groups according to the cut-off value of ALI determined by the Youden Index. Kaplan–Meier (K–M) curves were performed and analyzed by Log-rank test. Cox regression analysis was used to analyze the independent association between ALI with the primary and secondary endpoints. Univariate and multivariate Cox regression models were constructed, and adjusted in the multivariate Cox model based on the variables which were considered clinically relevant or with *p*-values < 0.05 in the univariate analysis. BMI, Alb, and NLR were excluded due to their direct correlation with ALI. The adjusted hazard ratio (HR) and 95% confidence interval (CI) were calculated. A two-sided *p*-value < 0.05 is regarded as statistical significance. HR >1.0 with a *p* < 0.05 indicated a deleterious effect while HR <1.0 with *p* < 0.05 indicated a protective effect. Net reclassification improvement (NRI) was computed when ALI was added to a base model in which the variables were statistically significant in the univariate analysis. Data were analyzed with SPSS version 25.0 (IBM, Armonk, NY, USA) and R version 4.2.1 (R Foundation for Statistical Computing, Vienna, Austria).

## 3. Results

### 3.1 Analysis of Baseline Data

From January 2013 to September 2020, a total of 245 consecutive patients were diagnosed with AMI complicated by CS. Of those, 28 patients were excluded due to incomplete data, leaving 217 patients for inclusion in the present study. The mean age of this cohort was 70.2 years, 65.0% was male, and the mean BMI was 23.1 ${\mathrm{kg/m}}^{2}$. Common comorbidities included hypertension (109, 50.2%) and diabetes mellitus (73, 33.6%). Prior PCI therapy was noted in eight patients. At admission, the mean SBP was 86 mmHg and mean heart rate was 88 bpm.

### 3.2 ALI ROC Analysis and Patient Categorization

**Fig. 2.**
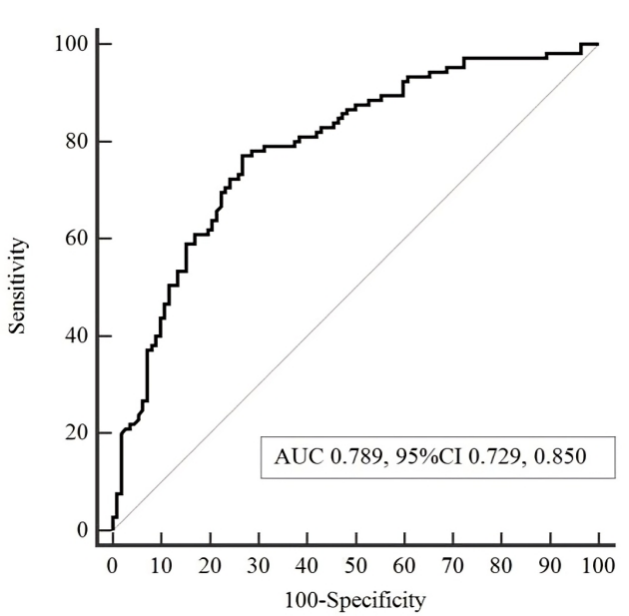
**Receiver operating characteristic (ROC) analysis of advanced lung cancer inflammation index (ALI) for predicting 30-day all-cause mortality.** AUC, area under the curve; CI, confidence interval.

This study aimed to assess the predictive capability of ALI in patients with AMI complicated by CS. The ROC curve analysis of ALI for predicting 30-day all-cause mortality was presented in Fig. 2. The ALI ROC for predicting 30-day all-cause mortality showed a modest predictive value (AUC: 0.789, 95% CI: 0.729, 0.850). Next, the Youden Index was used to determine the optimal cut-off value for ALI (12.69, with a sensitivity of 76.9% and a specificity of 72.6%). Subsequently, patients were categorized into two groups based on the ALI score: the low-ALI (≤12.69) and high-ALI (>12.69) groups (Table 1). Notably, the low-ALI group comprised older individuals with lower BMIs (all *p* < 0.001). In terms of vital signs at admission, the low-ALI group had a higher heart rate (mean 92 vs. 83 bpm, *p* = 0.012), while SBP and diastolic blood pressure showed no statistical difference. Regarding the medical history, the rate of heart failure was higher in the low-ALI group (*p* = 0.037). All other histories were comparable between the two groups (all *p* > 0.05).

**Table 1.** **Baseline patient characteristics based on the cut-off value of ALI**.

Baseline Characteristics		ALI ≤12.69	ALI >12.69	*p* value
		n = 111	n = 106	
Age (years)		72.92 ± 10.44	67.39 ± 11.72	<0.001
Male (%)		68 (61.3)	73 (68.9)	0.242
BMI (${\mathrm{kg/m}}^{2}$)		22.36 ± 2.91	23.91 ± 2.84	<0.001
Smoking (%)		56 (50.5)	58 (54.7)	0.531
Alcohol drinking (%)		33 (29.7)	38 (35.8)	0.339
Admission vital signs				
	SBP (mmHg)	87.34 ± 13.53	83.61 ± 14.80	0.054
	DBP (mmHg)	57.14 ± 11.23	54.44 ± 11.64	0.083
	Heart rate (bpm)	92.27 ± 26.80	82.69 ± 28.82	0.012
Medical histories (%)				
	Hypertension	59 (53.2)	50 (47.2)	0.381
	Diabetes mellitus	39 (35.1)	34 (32.1)	0.635
	COPD	6 (5.4)	4 (3.8)	0.569
	Dyslipidemia	7 (6.3)	8 (7.5)	0.720
	Stroke	8 (7.2)	7 (6.6)	0.862
	Previous CHD	20 (18.0)	20 (18.9)	0.873
	Previous MI	7 (6.3)	5 (4.7)	0.611
	Previous PCI	11 (9.9)	7 (6.6)	0.380
	Previous CABG	2 (1.8)	1 (0.9)	0.590
	Heart failure	9 (8.1)	2 (1.9)	0.037

ALI, advanced lung cancer inflammation index; CABG, coronary artery bypass grafting; CHD, coronary heart disease; COPD, chronic obstructive pulmonary disease; DBP, diastolic blood pressure; MI, myocardial infarction; PCI, percutaneous coronary intervention; SBP, systolic blood pressure; n, the number of patients; BMI, body mass index.

### 3.3 Patient Laboratory Parameters and Treatments

The admission laboratory parameters and administered treatments are displayed in Table 2. The low-ALI group showed increased levels of lactate, D-dimer, brain natriuretic peptide (BNP), creatinine, leucocyte count, neutrophil count, NLR, activated partial prothrombin time, prothrombin time, and international normalized ratio (all *p* < 0.05). Procalcitonin (PCT), high sensitivity C-reactive protein (hs-CRP), and cardiac troponin I (cTnI) were comparable between the two groups (all *p* > 0.05). On echocardiography examination, the low-ALI group had a lower left ventricular ejection fraction (LVEF) (mean 46.70% vs. 50.41%, *p* = 0.006). There were nonsignificant frequency trends, particularly anterior myocardial infarction was more common in the low-ALI group (38.7% vs. 34.0%, respectively), while inferior myocardial infarction was more common in the high-ALI group (36.9% vs. 50.0%, respectively). It is important to note that neither of these results reached statistical significance (all *p* > 0.05). Furthermore, patients in the low-ALI group were less likely to undergo coronary revascularization (55.9% vs. 79.2%, *p* < 0.001) and were less likely to be treated with aspirin, statins, beta-blocker, ACEI/ARB, and aldosterone antagonists (all *p* < 0.05). Conversely, this group had a higher reliance on mechanical ventilation (*p* = 0.002).

**Table 2.** **A comparison of laboratory parameters and treatment of the 2 groups based on the cut-off value of ALI**.

		ALI ≤12.69	ALI >12.69	*p* value
		n = 111	n = 106	
Lactate (mmol/L)		5.29 ± 3.44	4.03 ± 4.21	0.016
D-dimer		3.43 ± 6.31	1.36 ± 1.79	0.001
cTnI (ng/mL)		9.09 ± 10.12	6.79 ± 8.27	0.069
BNP (pg/mL)		2003.77 ± 1671.9	972.27 ± 1354.37	<0.001
Creatinine (µmol/L)		174.89 ± 109.15	106.51 ± 58.38	<0.001
hs-CRP (mg/L)		14.89 ± 5.36	14.25 ± 7.11	0.538
PCT (ng/mL)		8.04 ± 32.61	4.45 ± 23.84	0.446
Leucocyte (×${\mathrm{10}}^{9}$/L)		14.76 ± 5.36	8.39 ± 2.98	<0.001
Neutrophil (%)		86.47 ± 5.93	69.40 ± 7.30	<0.001
Lymphocyte (%)		7.47 ± 3.24	20.68 ± 7.30	<0.001
NLR		15.21 ± 11.39	3.77 ± 1.49	<0.001
APTT		39.52 ± 24.83	28.65 ± 7.82	<0.001
INR		1.51 ± 1.41	1.08 ± 0.17	0.002
PT		17.11 ± 12.14	12.54 ± 1.70	<0.001
Albumin (g/dL)		31.49 ± 6.81	36.65 ± 4.83	<0.001
LVEF (%)		46.70 ± 9.61	50.41 ± 9.83	0.006
Types of MI (n, %)				
	STEMI	92 (82.9)	92 (86.8)	0.423
	NSTEMI	19 (17.1)	14 (13.2)	0.423
MI localization on ECG (n, %)				
	Anterior MI	43 (38.7)	36 (34.0)	0.467
	Inferior MI	41 (36.9)	53 (50.0)	0.053
	Lateral MI	10 (9.0)	8 (7.5)	0.698
	Right Ventricular MI	12 (10.8)	15 (14.2)	0.458
Treatment (n, %)				
	Aspirin	98 (88.3)	102 (96.2)	0.030
	Clopidogrel	58 (52.3)	56 (52.8)	0.932
	Ticagrelor	53 (47.7)	62 (58.5)	0.113
	Beta-Blocker	53 (47.7)	70 (66.0)	0.007
	ACEI/ARB	27 (24.3)	47 (44.3)	0.002
	Diuretics	60 (54.1)	58 (54.7)	0.922
	Aldosterone Antagonist	18 (16.2)	30 (28.3)	0.032
	Statin	97 (87.4)	103 (97.2)	0.007
	Anticoagulant	31 (27.9)	26 (24.5)	0.569
	Dopamine/Norepinephrine	60 (54.1)	55 (51.9)	0.749
	Coronary Revascularization	62 (55.9)	84 (79.2)	<0.001
	IABP	17 (15.3)	19 (17.9)	0.606
	VA-ECMO	3 (2.7)	1 (0.9)	0.622
	Mechanical ventilation	61 (55.0)	36 (34.0)	0.002

ACEI, angiotensin-converting enzyme inhibitor; APTT, activated partial thromboplastin time; ARB, angiotensin receptor blocker; VA-ECMO, veno-arterial extracorporeal membrane oxygenation; BNP, brain natriuretic peptide; ECG, electrocardiogram; hs-CRP, high-sensitivity C-reactive protein; IABP, intra-aortic balloon pump; INR, international normalized ratio; LVEF, left ventricular ejection fraction; MI, myocardial infarction; NLR, neutrophil-lymphocyte ratio; NSTEMI, non-ST elevation myocardial infarction; PT, prothrombin time; STEMI, ST elevation myocardial infarction; cTnI, cardiac troponin I; ALI , advanced lung cancer inflammation index; PCT, procalcitonin; n, the number of patients.

### 3.4 Survival and Major Adverse Cardiovascular Events

**Fig. 3.**
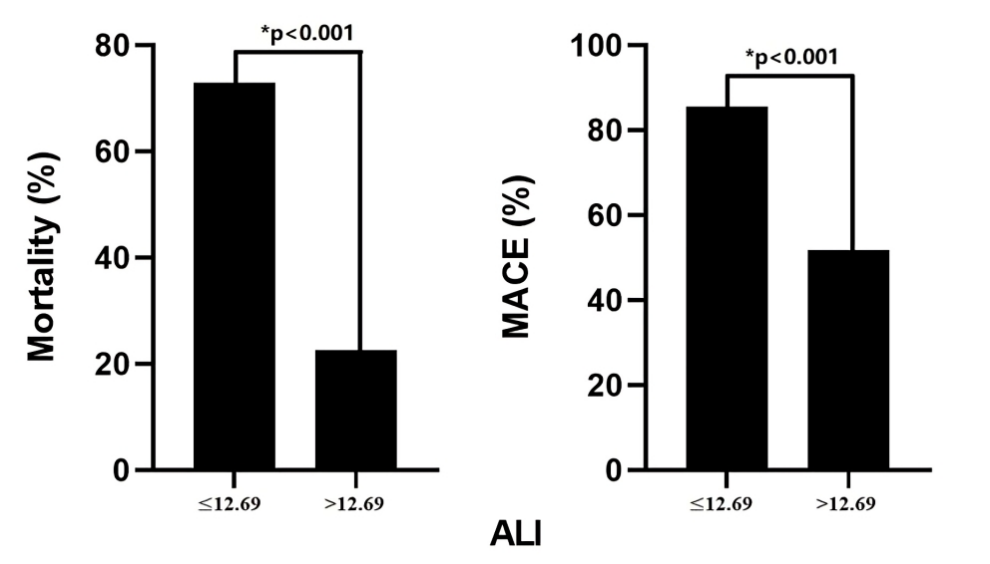
**Incidence of mortality and major adverse cardiovascular event (MACE) according to the cut-off value of advanced lung cancer inflammation index (ALI)**.

**Fig. 4.**
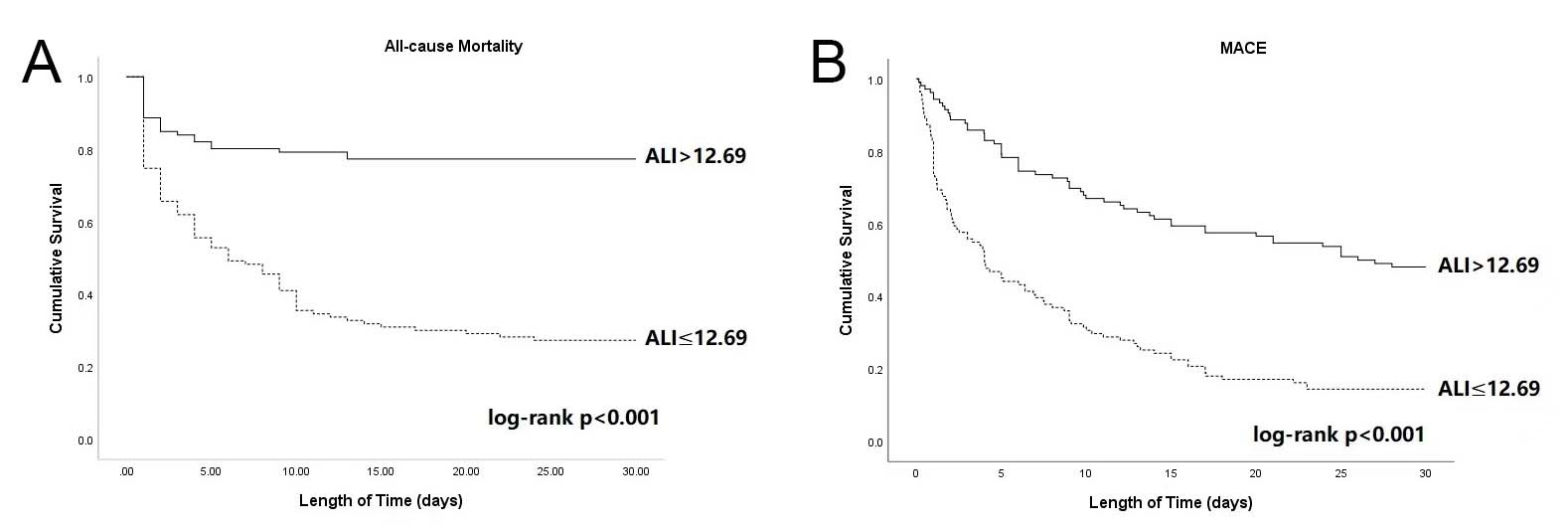
**Kaplan–Meier (K–M) curves of survival (A) and MACE (B) according to the cut-off value of ALI.** MACE, major adverse cardiovascular event; ALI, advanced lung cancer inflammation index.

During the 30-day follow-up period after admission, out of the total patient cohort 104 patients died (47.9%), including 80 patients in the low-ALI group and 24 patients in the high-ALI group. The 30-day mortality rate was significantly higher in the low-ALI group than in the high-ALI group (72.1% vs. 22.6%, *p* < 0.001). The secondary endpoint showed a higher rate of MACEs in the low-ALI group than in the high-ALI group (85.6% vs. 51.9%, *p* < 0.001) (Fig. 3). Fig. 4 shows the K–M curves of the two groups; the rates of cumulative mortality and free of MACEs in the low-ALI group were significantly higher than in the high-ALI group (both outcomes log-rank *p* < 0.001).

### 3.5 Factors Associated with Patient Survival

Cox regression was performed to determine the relationship between ALI and patient outcomes (Table 3). The univariate Cox proportional hazard analysis identified several factors associated with all-cause mortality, including age, sex, SBP, lactate, cardiac troponin, creatinine, LVEF, and ALI ≤12.69 (all *p* < 0.05). Notably, age, lactate, creatinine, LVEF, and ALI ≤12.69 were associated with 30-day MACEs (all *p* < 0.05). Furthermore, the multivariate Cox proportional hazard analysis revealed that an ALI score ≤12.69 was an independent predictor for both 30-day all-cause mortality (HR: 3.327; 95% CI: 2.053, 5.389; *p* < 0.001) and 30-day MACEs (HR: 2.250; 95% CI 1.553, 3.260; *p* < 0.001). Additional independent factors for 30-day all-cause mortality were age, SBP, lactate, and LVEF, while lactate, creatinine, and LVEF were independent risk factors for 30-day MACEs.

**Table 3.** **Cox regression models for the 30-day outcome**.

		Univariable		Multivariable	
		HR (95% CI)	*p* value	HR (95% CI)	*p* value
(A) 30-day Mortality					
	Age (1 year increase)	1.044 (1.025, 1.063)	<0.001	1.027 (1.007, 1.046)	0.007
	Male vs. Female	0.641 (0.434, 0.946)	0.025		
	Anterior MI	1.057 (0.712, 1.569)	0.783		
	Systolic blood pressure	0.987 (0.974, 0.999)	0.041	0.981 (0.966, 0.995)	0.010
	Lactate	1.095 (1.063, 1.128)	<0.001	1.074 (1.031, 1.119)	0.001
	Cardiac Troponin	1.021 (1.002, 1.041)	0.033		
	hs-CRP	1.018 (0.984, 1.052)	0.301		
	Procalcitonin	1.004 (0.997, 1.011)	0.269		
	Creatinine	1.003 (1.002, 1.005)	<0.001		
	LVEF	0.950 (0.932, 0.968)	<0.001	0.963 (0.943, 0.983)	<0.001
	ALI ≤12.69 (vs. ALI >12.69)	4.346 (2.743, 6.885)	<0.001	3.327 (2.053, 5.389)	<0.001
(B) MACE					
	Age (1 year increase)	1.024 (1.009, 1.039)	0.002		
	Male vs. Female	0.896 (0.640, 1.254)	0.522		
	Anterior MI	1.086 (0.779, 1.514)	0.627		
	Systolic blood pressure	0.994 (0.984, 1.004)	0.245		
	Lactate	1.073 (1.045, 1.102)	<0.001	1.069 (1.035, 1.104)	<0.001
	Cardiac Troponin	1.004 (0.987, 1.021)	0.664		
	hs-CRP	1.018 (0.991, 1.045)	0.191		
	Procalcitonin	1.002 (0.996, 1.009)	0.483		
	Creatinine	1.005 (1.003, 1.006)	<0.001	1.002 (1.001, 1.004)	<0.001
	LVEF	0.967 (0.951, 0.983)	<0.001	0.980 (0.963, 0.996)	0.018
	ALI ≤12.69 (vs. ALI >12.69)	2.878 (2.054, 4.032)	<0.001	2.250 (1.553, 3.260)	<0.001

ALI, advanced lung cancer inflammation index ratio; hs-CRP, high-sensitivity C-reactive protein; LVEF, left ventricular ejection fraction; MACE, major adverse cardiovascular events; MI, myocardial infarction. HR, hazard ratio; CI, confidence interval.

To evaluate the incremental predictive value of ALI, a base model was built based using the variables that were statistically significant in the univariate Cox proportional hazard analysis. The base model of 30-day all-cause mortality included age, sex, lactate, cTnI, creatinine, and LVEF (AUC: 0.870; 95% CI: 0.824, 0.916). The base model of 30-day MACEs included age, lactate, creatinine, and LVEF (AUC: 0.787; 95% CI: 0.725, 0.849). The addition of the ALI to the new model resulted in a significant improvement in risk stratification for 30-day all-cause mortality (AUC: 0.901, 95% CI: 0.860, 0.941; categorical NRI: 0.659, 95% CI: 0.462, 0.788, *p* < 0.001; continuous NRI: 1.685; 95% CI: 1.211, 1.847, *p* < 0.001) and 30-day MACEs (AUC: 0.805, 95% CI: 0.746, 0.865; categorical NRI: 0.430, 95% CI: 0.220, 0.722, *p* < 0.001; continuous NRI: 1.213, 95% CI: 0.912, 1.414, *p* < 0.001).

## 4. Discussion

In this study, we evaluated ALI, an inflammatory index, as a prognostic marker in patients with AMI complicated by CS. We demonstrated that patients with a low ALI had less favorable short-term outcomes, suggesting that ALI could serve as a prognostic tool in this patient population. To the best of our knowledge, this is the first study to examine the prognostic value of ALI in patients with AMI complicated by CS.

### 4.1 ALI and Inflammation

Originally, ALI was developed to assess the degree of systemic inflammation in patients with metastatic NSCLC, where an ALI <18 indicated a poor prognosis [6]. Further studies found that ALI could serve as a prognostic marker in patients with other diseases [7, 8]. ALI is calculated based on the BMI, Alb, and NLR, making it a unique index that encapsulates both nutritional and inflammatory aspects. Considering the important role of inflammation and nutrition in cardiovascular disease, ALI may have important prognostic value. In fact, previous studies have demonstrated that inflammation activation is involved in the pathogenesis of cardiovascular disease and inflammatory markers have been shown to be associated with the outcome [12]. Nutritional status reflects a patient’s general condition, including physical condition, protein turnover, and immune competence; therefore, nutritional status has become increasingly important in patients with cardiovascular disease [13]. However, ALI has not been widely recognized as an inflammatory and nutritional marker. Until now, studies on the association of ALI with the outcome in patients with cardiovascular diseases remain scarce, with only two studies conducted in patients with heart failure and hypertension.

### 4.2 Current Evidence Regarding the Prognostic Role of ALI in Cardiovascular Diseases

In a study by Maeda *et al*. [9] patients with acute decompensated heart failure in the lowest tertile of ALI were found to have the highest all-cause mortality and readmission rates. Similarly, Yuan *et al*. [14] explored the prognostic value of ALI in elderly patients with heart failure and found that during a median follow-up of 28 months, ALI was an independent predictor for all-cause mortality and cardiovascular mortality. Furthermore, in patients with hypertension, a high ALI was associated with a reduced risk of cardiovascular death [15, 16]. Despite these findings, research exploring the association of ALI with the outcome in patients with AMI complicated by CS remains scarce.

Our study fills this gap by demonstrating that a low ALI is correlated with increased risk of short-term all-cause mortality and MACEs in patients with AMI complicated by CS. This suggests that patients with a lower ALI have a more severe inflammatory status than those with higher ALI. Our findings extend previous research, affirming ALI’s prognostic value in AMI cases complicated by CS.

It is worth noting that there was no universal cut-off value for ALI, and the cut-off value in the present study is lower than in other studies, possibly due to the different patients enrolled. Our present study focused on patients with AMI complicated by CS, indicatingthese individuals may experience more severe myocardial impairment and inflammation compared to those with conditions like heart failure or hypertension.

### 4.3 Possible Mechanisms of ALI as a Novel Biomarker in AMI Complicated by CS

The mechanism behind the association between ALI and the prognosis of patients with AMI complicated by CS may be related to the components involved in this index. Previous studies have shown that BMI [17] and Alb [18, 19, 20], as nutritional factors, are associated with the outcome of patients with AMI and CS. In addition, present study found that patients with a low ALI presented with a relatively lower BMI and Alb. NLR an increasingly recognized marker in inflammatory diseases including cardiovascular disease, reflects inflammation activation, which is notably more severe in CS following AMI. Our previous study has confirmed the prognostic value of NLR in patients with AMI complicated by CS [4].

Meanwhile, markers like hs-CRP and PCT are used to assess inflammatory activation, in our study, their levels were similar across groups. This suggests that leukocytes and their subgroups might be more effective indicators in this context.

Previous studies have linked the degree of inflammation to the severity of myocardial infarction [21, 22], indicating that a higher NLR is a marker of larger infarction size, as reflected by an elevated cTnI level and reduced LVEF in patients with ALI ≤12.69. Additionally, in the ALI ≤12.69 group, lactate, D-dimer, BNP, and coagulation indices were significantly elevated, which were all risk factors of poor outcome in patients with AMI complicated by CS. However, after multivariable adjustment, ALI was an independent factor for short-term outcomes, suggesting its independent predictive value. Therefore, ALI, as a combined inflammatory and nutritional marker, was associated with the prognosis of patients with AMI complicated by CS.

A key finding of our study is the enhanced predictive ability of traditional Cox models by integrating the ALI. This improvement highlights the potential of ALI, an easily accessible index, in enhancing risk stratification. Given the complexity of CS and the critical importance of accurate risk stratification for prognostic assessment, incorporating ALI could be particularly valuable.

Last but not least, ALI had the highest HR value among the variables considered in the multivariable model. Coupled with its area under the ROC curve value, these findings underscore the significance of ALI as a critical prognostic marker. Its integration into existing risk assessment models could significantly enhance the evaluation and management of patients with AMI complicated by CS.

## 5. Limitations

There are some potential limitations that should be addressed in this study. First, this was a single-center, retrospective study with a relatively small sample size, which may limit the generalizability of the findings. Second, ALI is a composite index and the variables used in its calculation are not specific or limited to any disease; thus, they may be influenced by various factors including nutritional status, chronic inflammation, and medication. Third, our analysis focused solely on the prognostic value of ALI and its components. Other inflammatory markers, such as interleukin-1, interleukin-6, and tumor necrosis factor-α, which could also have significant prognostic implications, were not assessed. Fourth, mechanical circulatory support devices, such as Impella and extracorporeal membrane oxygenation can be used to maintain organ perfusion and oxygenation. Although there is evidence their usage can improve outcomes [23], these devices were not commonly used in our center during the enrolled period. Fifth, we only used ALI measurements taken upon admission for this study. Given that inflammatory and nutritional status can change rapidly, a series of tests may provide a more comprehensive understanding of their association with patient outcomes. In addition, the small sample size of our study influenced the determination of the ALI cut-off value. Comparisons of ALI among different groups, such as a normal population or AMI patients without complications from CS, would be beneficial to further understand the extent of ALI changes in the context of AMI complicated by CS. Consequently, these results should be interpreted with caution, and additional studies with larger sample sizes are necessary to validate our findings.

## 6. Conclusions

Upon admission, assessing ALI levels can provide important information for the short-term prognostic assessment of patients with AMI complicated by CS. A lower ALI may serve as an independent predictor of 30-day all-cause mortality and MACEs.

## Data Availability

All data relevant to the study are included in the article or uploaded as supplementary files. Data can also be requested from the corresponding author.
